# Significance of inflammation-based indices in the prognosis of patients with non-metastatic colorectal cancer

**DOI:** 10.18632/oncotarget.16774

**Published:** 2017-04-01

**Authors:** Xiangping Song, Hong Zhu, Qian Pei, Fengbo Tan, Chenglong Li, Zhongyi Zhou, Yuan Zhou, Nanhui Yu, Yuqiang Li, Haiping Pei

**Affiliations:** ^1^ Department of Gastrointestinal Surgery, Xiangya Hospital, Central South University, Changsha, China; ^2^ Department of Oncology, Xiangya Hospital, Central South University, Changsha, China

**Keywords:** colorectal cancer, prognostic factors, inflammation, survival, coNLR-PDW

## Abstract

Previous studies demonstrated that several inflammation-based hematological indices are closely related to various malignancies, including colorectal cancer (CRC). In this study, the prognostic value of inflammation-based markers, including a combination index termed coNLR-PDW, comprising the preoperative neutrophil-to-lymphocyte ratio (NLR) and the platelet distribution width (PDW), was evaluated in 206 patients with non-metastatic CRC treated with surgery at a single medical center. The association of patient demographics, blood chemistry, and serum biochemical indices with recurrence-free survival (RFS) and overall survival (OS) were examined through univariate and multivariate analysis. Receiver operating characteristic curve analysis revealed the optimal cut-off values of the NLR and lymphocyte-to-monocyte ratio (LMR) to be, respectively, 2.0 and 3.32 for both RFS and OS. For PDW, cut-off values of 17.25% and 17.35% were defined for RFS and OS, respectively. On univariate analysis, lymph node involvement, stage, presence of intravascular emboli (IVE), carbohydrate antigen 199 (CA199) ≥ 35 kU/L, NLR ≥ 2.0, LMR ≤ 3.32, elevated PDW, a high coNLR-PDW score, high blood glucose, and high neutrophil and lymphocyte percentages correlated with poorer RFS and OS (*P* < 0.05). On multivariate analysis, lymph node involvement, IVE, CA199, PDW, and coNLR-PDW correlated with both RFS and OS (*P* < 0.05), while NLR correlated only with OS (*P* = 0.001). These results highlight the usefulness of the coNLR-PDW index as a prognostic marker of non-metastatic CRC outcome. In clinical practice, its assessment could contribute to establishing more personalized regimes for patients undergoing tumor resection surgery.

## INTRODUCTION

Colorectal cancer (CRC) is one of the most common malignancies worldwide, ranking third and fourth, respectively, in cancer-related morbidity and mortality. In contrast with Western countries, the incidence of CRC in China is rising continuously [1, 2].

The tumor-node-metastasis (TNM) stage system can predict the prognosis of CRC and many other cancers, and contributes to a great extent to directing the treatment of CRC. In non-metastatic colon cancer, 5-years survival rates range from 58.3% to 82.7% [3], however, clinical outcomes vary considerably among patients with the same TNM stage [4]. Recently, other factors such as microsatellite instability (MSI), the state of KRAS and BRAF, and tumor location have been added to supplement the TNM stage system with the goal of improving prognosis prediction and helping guide clinical therapies [5]. Still, these prognostic markers are often insufficient to accurately predict CRC prognosis.

The association between inflammation and cancer has been widely confirmed since it was first proposed by Virchow in 1863 [6]. Ever since, several inflammation-based prognostic systems have been established, such as the Glasgow Prognostic Score (GPS), the systemic inflammation score (SIS), the neutrophil to lymphocyte ratio (NLR), the lymphocyte to monocyte ratio (LMR), platelet count, among others [7–11], many of which focus on the status of blood cells involved in inflammatory reactions. In clinical studies, decreased LMR [9], increased platelet count, and increased NLR [10, 12] were all related to inferior survival in patients with CRC. Some of the mechanisms by which leukocytes and platelet affect tumor proliferation and invasion have been elucidated [13–14]. In addition, recent findings have confirmed that aspirin, an anti-platelet drug, can reduce CRC incidence and mortality and its use has been suggested in individuals with high CRC risk factors [15].

It has long been known that while activated platelets play a key role in inflammation, they typically constitute a minor fraction of the total platelet population. For this reason, and despite many recent studies implying so, platelet count can't accurately represent platelet activity as an indicator of inflammation. Instead, since platelet size reflects platelet activity, the latter can be assessed by platelet volume indices (PVI) including mean platelet volume (MPV), platelet distribution width (PDW), and platelet large cell ratio (P-LCR) [16]. In this report we assessed the prognostic utility of several blood indices, which can be easily and inexpensively estimated, in patients with non-metastatic CRC who underwent radical resection. The present study is one of the few that focused on PDW and the first, to our knowledge, to assess the prognostic value of the coNLR-PDW index in non-metastatic CRC patients.

## RESULTS

### Patients’ characteristics

A total of 206 patients with non-metastatic CRC that received resective surgery in our Hospital between January 2009 and December 2011 were enrolled in the study. The ratio of male to female was around 1.5:1. Among all patients, 106 (51.5%) had colon cancer and 100 (48.5%) had rectal cancer. The distribution by CRC stage was 37 (18.0%) with stage I, 67 (32.5%) with stage II, and 102 (49.5%) with stage III CRC (Supplementay Table 1). The mean age and age range at the time of diagnosis were 57 years and 23 to 83 years, respectively. Laboratory results, including various blood cell counts, platelet volume indices, coagulation-related indices, etc., are shown in Supplementay Table 1. The last date of follow-up was November 15, 2015 and the median follow-up duration was 52 months (range from 3 to 82 months).

**Figure 1:**
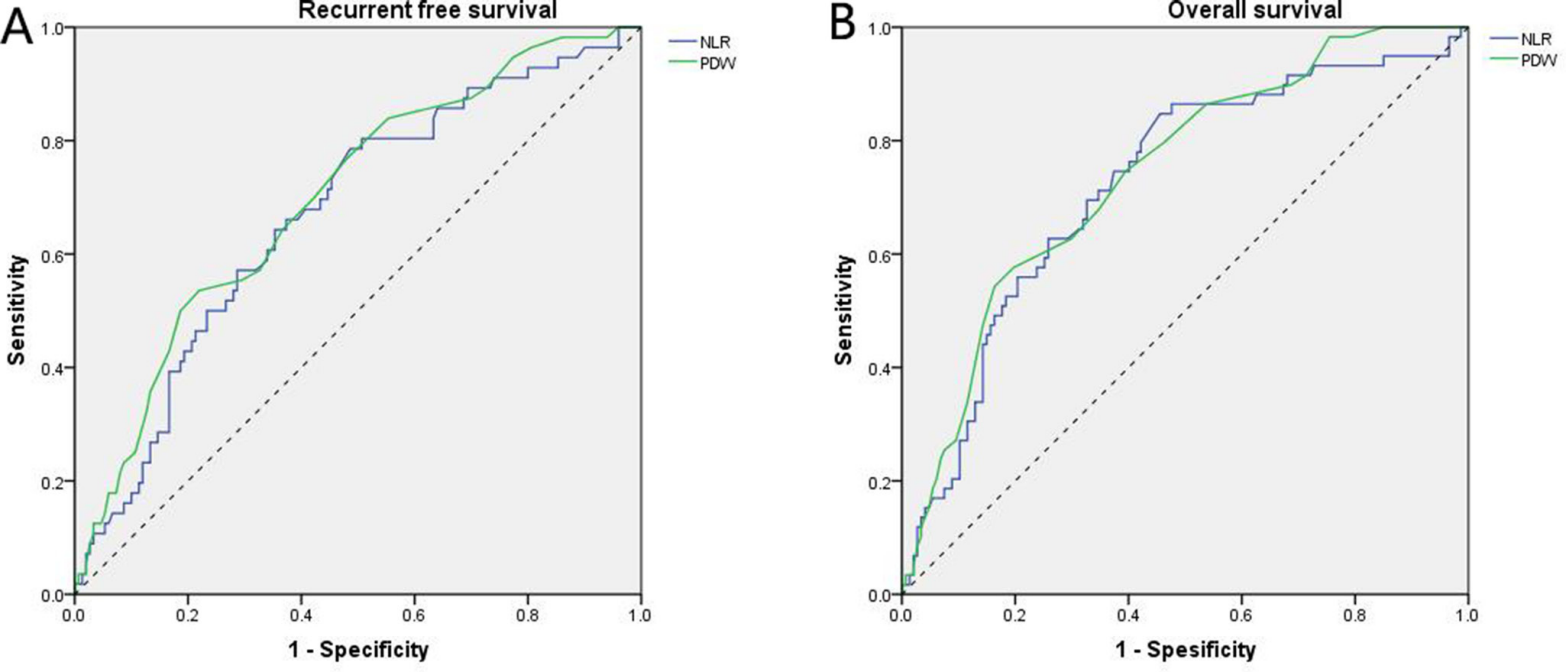
Receiver operating characteristic curve analyses (**A**) For RFS, NLR is represented by the blue line with an area under the curve (AUC) = 0.670 (95%CI, 0.589–0.752, *P* < 0.001) with a sensitivity of 78.6% and a specificity of 51.3%, and PDW is represented by the green line with an AUC = 0.700 (95%CI, 0.621–0.779, *P* < 0.001) with a sensitivity of 53.6% and a specificity of 78.0%; (**B**) For OS, NLR is represented by the blue line with an AUC = 0.724 (95%CI, 0.647–0.801, *P* < 0.001) with a sensitivity of 84.7% and a specificity of 54.6%, and PDW is represented by the green line with an AUC = 0.743 (95%CI, 0.0.671–0.815, *P* < 0.001) with a sensitivity of 54.2% and a specificity of 83.7%.

Applying receiver operating characteristic curve (ROC) analysis, the cut-off values of PDW were defined at 17.25% for RFS and 17.35% for OS. For NLR, the optimal cut-off value was 2.0 for both RFS and OS (Figure 1), while a LMR cut-off value of 3.32 was defined for both RFS and OS (data not shown).

Table 1 shows the distribution of the clinical background characteristics of the studied patients grouped according to PDW and NLR. After segregating by PDW (with cut-off set at 17.35%), significant inter-group differences were found for TNM stage (*P* = 0.035), lymph node involvement (*P* = 0.010), intravascular emboli (IVE; *P* = 0.013), and LMR (*P* = 0.016). After separation by NLR, significant differences in tumor differentiation (*P* = 0.001), IVE (*P* = 0.001), and LMR (*P* < 0.001) were detected between groups. In addition, significant, albeit more modest, differences between these groups were also found for factors such as gender, tumor invasion depth, and tumor size. We also analyzed the distribution differences between groups defined by PDW (with 17.25% as the cut-off value), and significant differences were only detected in lymph node involvement (*P* = 0.018) and IVE (*P* = 0.034) (Supplementay Table 2).

**Table 1:** Relationships between clinical characteristics and PDW or NLR

Parameters	PDW (%)		*P*	NLR		*P*
	PWD < 17.35	PDW ≥ 17.35		NLR < 2.0	NLR ≥ 2.0	
	(*n* = 150)	(*n* = 56)		(*n* = 87)	(*n* = 119)	
Age						
≥ 60	65	28	0.392	34	59	0.135
< 60	85	28		53	60	
Gender						
Male	87	38	0.198	46	79	**0.050**
Female	63	18		41	40	
Location						
Colon	75	31	0.494	42	64	0.481
Rectum	75	25		45	55	
Stage						
TNM I	30	7	**0.035**	19	18	0.464
TNM II	54	13		27	40	
TNM III	66	36		41	61	
Differentiation						
Well	119	43	0.691	78	84	**0.001**
Poor	31	13		9	35	
Tumor invasion depth						
T1–2	37	15	0.755	28	24	**0.050**
T3–4	113	41		59	95	
Lymph node involvement						
N0	84	20	**0.010**	46	58	0.558
N+	66	36		41	61	
IVE						
Absence	119	35	**0.013**	12	40	**0.001**
Presence	31	21		75	79	
Diameter						
≥ 5 cm	89	30	0.456	43	76	**0.046**
< 5 cm	61	26		44	43	
CEA						
≥ 5 ng/ml	37	13	0.829	21	29	0.969
< 5 ng/ml	113	43		66	90	
CA199						
≥ 35 kU/L	20	10	0.413	12	18	0.789
< 35 kU/L	130	46		75	101	
LMR						
≥ 3.32	95	25	**0.016**	73	47	**< 0.001**
< 3.32	55	31		14	72	
NLR						
≥ 2.0	82	37	0.140			NA
< 2.0	68	19				
Adjuvant chemotherapy						
Yes	91	28	0.168	52	67	0.619
No	59	28		35	52	

Abbreviations: IVE, intravascular emboli; CEA, carcinoembryonic antigen; CA199, carbohydrate antigen 199; LMR, lymphocyte to monocyte ratio; NLR, neutrophil to lymphocyte ratio; PDW, platelet distribution width.

Table 2 shows the clinicolaboratory characteristics of the groups defined above. Significant inter-group differences were found for lymphocyte percentage (*P* = 0.018), MPV (*P* < 0.001), LMR (*P* = 0.019), and PDW (*P* < 0.001) when patients were grouped by PDW (17.35% as the cut-off value). When patients were grouped by NLR, significantly differences were found for age (*P* = 0.007), WBC count (*P* < 0.001), neutrophil percentage (*P* < 0.001), lymphocyte percentage (*P* < 0.001), NLR (*P* = 0.007), and LMR (*P* < 0.001). Similar results were obtained when patients were grouped by PDW with a cut-off value of 17.25% (Supplementay Table 3).

**Table 2:** Relationships between clinicolaboratory characteristics and PDW or NLR

Parameters	PDW (%)		*P*	NLR		*P*
	PWD < 17.35	PDW ≥ 17.35		NLR < 2.0	NLR ≥ 2.0	
	(*n* = 150)	(*n* = 56)		(*n* = 87)	(*n* = 1 19)	
Age (year)	56.7 ± 12.6	58.0 ± 12.7	0.514	54.3 ± 11.9	59.1 ± 12.8	**0.007**
Albumin (g/dl)	38.1 ± 3.7	38.4 ± 4.4	0.591	38.4 ± 3.4	38.0 ± 4.2	0.548
Blood glucose (mmol/L)	5.0 ± 1.0	5.0 ± 1.2	0.780	4.9 ± 0.9	5.1 ± 1.1	0.130
Platelet count (×10^3^/ml)	249.0 ± 94.9	221.5 ± 81.1	0.056	227.7 ± 73.7	251.7 ± 102.5	0.064
Hemoglobin (g/L)	112.7 ± 22.3	111.2 ± 23.3	0.683	114.7 ± 21.4	110.5 ± 23.3	0.188
WBC count (×10^3^/ml)	6.2 ± 2.0	5.8 ± 2.2	0.193	5.4 ± 1.4	6.6 ± 2.3	**< 0.001**
RBC count (×10^6^/ml)	4.0 ± 0.5	4.0 ± 0.6	0.388	4.0 ± 0.5	4.0 ± 0.6	0.180
Neutrophil percentage (%)	59.4 ± 9.5	62.0 ± 9.4	0.093	51.8 ± 5.5	66.1 ± 6.9	**< 0.001**
Lymphocyte percentage (%)	28.5 ± 8.4	25.3 ± 8.7	**0.018**	35.0 ± 5.7	22.3 ± 6.0	**< 0.001**
Monocyte percentage (%)	7.8 ± 2.4	8.5 ± 2.5	0.057	8.2 ± 2.2	7.8 ± 2.6	0.232
MPV (fl)	8.4 ± 1.5	9.7 ± 1.7	**< 0.001**	8.9 ± 1.5	8.7 ± 1.7	0.437
PT (sec)	12.8 ± 0.8	12.9 ± 0.7	0.251	12.7 ± 0.7	12.9 ± 0.8	0.138
INR	0.97 ± 0.08	0.99 ± 0.08	0.100	0.97 ± 0.07	0.98 ± 0.08	0.232
APTT (sec)	36.2 ± 4.0	37.3 ± 3.3	0.084	36.4 ± 3.7	36.6 ± 3.9	0.765
NLR	2.6 ± 3.0	5.1 ± 17.3	0.280	1.5 ± 0.3	4.6 ± 12.2	**0.007**
LMR	4.1 ± 2.5	3.3 ± 1.6	**0.019**	4.6 ± 1.8	3.4 ± 2.5	**< 0.001**
PDW (%)	16.4 ± 1.2	18.1 ± 1.0	**< 0.001**	16.9 ± 1.0	16.9 ± 1.6	0.868

Abbreviations: WBC, white blood cell; RBC, red blood cell; MPV, mean platelet volume; PT, prothrombin time; INR, international normalized ratio; APTT, activated partial thromboplastin time.

Table 3 and Table 4 show the associations between clinicolaboratory characteristics and the three groups of patients separated according to the coNLR-PDW index scores (as defined in Mat & Methods). We found significant differences among the three groups in tumor differentiation (*P* = 0.009), IVE (*P* = 0.001), LMR (≥ 3.32/< 3.32, *P* < 0.001), age (*P* = 0.034), WBC count (*P* < 0.001), neutrophil percentage (*P* < 0.001), lymphocyte percentage (*P* < 0.001), and MPV (*P* = 0.008). However, NLR, LMR, and PDW, which served as either categorical data or measurement data, also showed significant differences among the three groups (*P* < 0.001).

**Table 3:** Relationships between clinical characteristics and coNLR-PDW

Parameters	coNLR-PDW 0	coNLR-PDW 1	coNLR-PDW 2	*P*
	*n* = 68	*n* = 101	*n* = 37	
Age				
≥ 60	26	47	20	0.276
< 60	42	54	17	
Gender				
Male	36	61	28	0.074
Female	32	40	9	
Location				
Colon	33	51	22	0.544
Rectum	35	50	15	
Stage				
TNM I	17	15	5	0.129
TNM II	22	37	8	
TNM III	29	49	24	
Differentiation				
Well	62	73	27	**0.009**
Poor	6	28	10	
Tumor invasion depth				
T1–2	22	21	9	0.235
T3–4	46	80	28	
Lymph node involvement				
N0	39	52	13	0.090
N+	29	49	24	
IVE				
Absence	7	29	16	**0.001**
Presence	61	72	21	
Diameter				
≥ 5 cm	33	66	20	0.083
< 5 cm	35	35	17	
CEA				
≥ 5 ng/ml	14	30	6	0.180
< 5 ng/ml	54	71	31	
CA199				
≥ 35kU/L	9	14	7	0.704
< 35kU/L	59	87	30	
LMR				
≥ 3.32	59	50	11	**< 0.001**
< 3.32	9	51	26	
NLR				
≥ 2.0	0	82	37	**< 0.001**
< 2.0	68	19	0	
PDW				
≥ 17.35%	0	19	37	**< 0.001**
< 17.35%	68	82	0	
Adjuvant chemotherapy				
Yes	42	59	18	0.422
No	26	42	19	

**Table 4:** Relationships between clinicolaboratory characteristics and coNLR-PDW

Parameters	coNLR-PDW 0(*n* = 68)	coNLR-PDW 1(*n* = 101)	coNLR-PDW 2(*n* = 37)	*P*
Age (year)	53.8 ± 11.9	58.5 ± 12.7	59.0 ± 13.0	**0.034**
Albumin (g/dl)	38.3 ± 3.5	38.0 ± 3.7	38.2 ± 3.9	0.849
Blood glucose (mmol/L)	4.9 ± 1.0	5.0 ± 1.0	5.2 ± 1.3	0.459
Platelet count (×10^3^/ml)	235.4 ± 75.3	249.0 ± 103.1	241.5 ± 92.0	0.515
Hemoglobin (g/L)	114.7 ± 20.9	111.7 ± 23.9	109.4 ± 23.0	0.489
WBC count (×10^3^/ml)	5.4 ± 1.4	6.6 ± 2.1	6.1 ± 2.5	**< 0.001**
RBC count (×10^6^/ml)	4.0 ± 0.5	4.0 ± 0.5	4.0 ± 0.7	0.813
Neutrophil percentage (%)	51.9 ± 5.8	63.0 ± 8.7	67.2 ± 6.4	**< 0.001**
Lymphocyte percentage (%)	35.2 ± 6.1	25.1 ± 7.0	20.7 ± 6.3	**< 0.001**
Monocyte percent (%)	8.1 ± 2.3	7.7 ± 2.4	8.4 ± 2.8	0.279
MPV (fl)	8.5 ± 1.2	8.7 ± 1.7	9.5 ± 1.7	**0.008**
PT (sec)	12.7 ± 0.7	12.9 ± 0.8	12.9 ± 0.8	0.116
INR	0.96 ± 0.07	0.99 ± 0.08	0.99 ± 0.08	0.096
APTT (sec)	36.2 ± 3.8	36.5 ± 3.9	37.2 ± 3.5	0.389
NLR	1.5 ± 0.3	3.1 ± 3.5	7.0 ± 21.2	**< 0.001**
LMR	4.8 ± 2.0	3.7 ± 2.6	2.8 ± 1.6	**< 0.001**
PDW (%)	16.5 ± 0.5	16.7 ± 1.6	18.1 ± 1.0	**< 0.001**

### Survival outcomes

In our study cohort, and during the follow-up period, 56 patients (27.2%) developed tumor recurrence. Among those, 8 showed local recurrence and 48 developed metastasis. 59 patients (29%) died, 2 from cardiovascular and cerebrovascular events, 1 in a traffic accident, 1 from chemotherapeutic toxicity, 51 from cancer recurrence, and the other 4 due to unknown reasons. Tumors recurred in 6 out of 68 patients (8.8%) with a coNLR-PDW score of 0, 28 out of 101 patients (27.7%) with a coNLR-PDW score of 1, and 22 out of 37 patients (59.5%) with a coNLR-PDW score of 2 (*P* < 0.001). Death occurred in 4 patients (5.9%) with a coNLR-PDW score of 0, 28 patients (27.7%) with a coNLR-PDW score of 1, and 27 patients (73.0%) with a coNLR-PDW score of 2 (*P* < 0.001).

Univariate and multivariate analyses were performed to evaluate the relationship between clinical characteristics and patients’ prognoses. By univariate analysis we found that lymph node involvement, TNM stage, IVE, carbohydrate antigen 199 (CA199), NLR, LMR, PDW, coNLR-PDW, blood glucose, neutrophil percentage, and lymphocyte percentage were all associated with both RFS and OS. Although tumor invasion depth showed a significant association with RFS (*P* = 0.023), it was not associated with OS. On the other hand, tumor location was associated with OS (*P* = 0.007), but not with RFS (Table 5). Because clinical TNM stage derives from tumor invasion depth (T) and lymph node involvement (N), T, N, and other factors, but not TNM stage, with *P* values < 0.05 in univariate analysis, were included in the COX multivariate model for further analysis. Both by univariate analysis and multivariate analysis, patients with IVE, lymph node involvement, high CA199 (≥ 35 kU/L), and high scores of coNLR-PDW had inferior RFS and OS, while patients with rectal cancer showed worse OS than those with colon cancer. In univariate, but not multivariate, analysis, high preoperative LMR (≥ 3.32) was associated with better RFS (HR: 0.382, 95%CI: 0.223–0.653; *P* < 0.001) and OS (HR: 0.322, 95%CI: 0.189–0.550; *P* < 0.001). Similar results were obtained for the neutrophil and lymphocyte percentages (Table 6).

**Table 5:** Univariate analysis in relation to RFS and OS

Parameters		RFS			OS		
		*P*	HR	95% CI	*P*	HR	95% CI
Age	≥ 60						
	< 60	0.435	0.811	0.479–1.372	0.068	0.619	0.370–1.035
Gender	Male						
	Female	0.885	1.040	0.611–1.772	0.484	0.827	0.485–1.049
Location	Colon						
	Rectum	0.116	1.528	0.901–2.590	**0.007**	2.062	1.215–3.498
Differentiation	Well						
	Poor	0.423	1.281	0.699–2.347	0.187	1.472	0.828–2.616
Tumor invasion depth	T1–2						
	T3–4	**0.023**	2.505	1.134–5.532	0.072	1.919	0.943–3.904
Lymph node invasion	N0						
	N+	**< 0.001**	3.769	2.056–6.909	**< 0.001**	4.408	2.379–8.167
Stage	TNM I	**< 0.001**			**< 0.001**		
	TNM II	0.006	4.771	1.578–14.420	0.147	3.053	0.676–13.783
	TNM III	< 0.001	4.391	2.267–8.505	0.001	10.232	2.481–42.193
Diameter	< 5 cm						
	≥ 5 cm	0.138	0.658	0.378–1.144	0.140	0.666	0.388–1.142
IVE	Absence						
	Presence	**< 0.001**	6.117	3.568–10.488	**< 0.001**	7.289	4.284–12.404
CEA	< 5 ng/ml						
	≥ 5 ng/ml	0.291	1.368	0.765–2.444	0.288	1.358	0.772–2.388
CA199	< 35 kU/L						
	≥ 35 kU/L	**< 0.001**	2.996	1.676–5.356	**0.008**	2.258	1.238–4.120
NLR	< 2.0						
	≥ 2.0	**< 0.001**	2.996	1.676–5.356	**< 0.001**	4.551	2.588–8.003
LMR	< 3.32						
	≥ 3.32	**< 0.001**	0.382	0.223–0.653	**< 0.001**	0.322	0.189–0.550
PDW	< 17.35%						
	≥ 17.35%	NA			**< 0.001**	3.878	2.321–6.478
PDW	< 17.25%						
	≥ 17.25%	**< 0.001**	3.324	1.911–5.476	NA		
coNLR-PDW	0	**< 0.001**			**< 0.001**		
	1	0.005	3.517	1.456–8.497	0.002	5.229	1.833–14.914
	2	< 0.001	10.485	4.235–25.960	< 0.001	19.534	6.818–55.969
Adjuvant chemotherapy	Yes						
	No	0.067	0.586	0.331–1.039	0.284	0.746	0.437–1.275
Age (year)		0.587	1.006	0.985–1.028	0.672	1.005	0.983–1.026
Albumin (g/dl)		0.645	0.984	0.917–1.055	0.986	1.001	0.934–1.072
Blood glucose (mmol/L)		**0.018**	1.266	1.042–1.538	**0.022**	1.250	1.033–1.512
Platelet count (×10^3^/ml)		0.616	0.999	0.996–1.002	0.414	0.999	0.996–1.002
Hemoglobin (g/L)		0.304	0.994	0.983–1.005	0.558	0.997	0.986–1.008
WBC count (×10^3^/ml)		0.091	0.877	0.753–1.021	0.418	0.945	0.823–1.084
RBC count (×10^6^/ml)		0.793	0.938	0.581–1.514	0.957	0.987	0.616–1.582
Neutrophil percentage (%)		**< 0.001**	1.047	1.021–1.074	**< 0.001**	1.055	1.029–1.080
Lymphocyte percentage (%)		**< 0.001**	0.947	0.920–0.974	**< 0.001**	0.935	0.909–0.962
Monocyte percent (%)		0.357	1.050	0.946–1.166	0.458	1.040	0.938–1.153
MPV (fl)		0.700	1.032	0.880–1.210	0.652	1.036	0.887–1.211
PT (sec)		0.423	1.138	0.830–1.561	0.765	1.050	0.764–1.443
INR		0.138	10.060	0.478–211.815	0.504	2.946	0.124–70.102
APTT (sec)		0.657	1.061	0.947–1.089	0.949	1.002	0.936–1.073
NLR		**0.017**	1.017	1.003–1.032	**0.005**	1.020	1.006–1.035
LMR		**0.010**	0.785	0.652–0.944	**0.002**	0.748	0.622–0.901
PDW (%)		**< 0.001**	1.442	1.202–1.731	**< 0.001**	1.510	1.268–1.798

**Table 6:** Multivariate analysis in relation to RFS and OS

Parameters	RFS			OS		
	*P*	HR	95% CI	*P*	HR	95% CI
Location (colon vs rectum)	NA			**< 0.00**1	2.923	1.621–5.301
Tumor invasion depth (T1–2 vs T3–4)	0.328	1.501	0.665–3.389	NA		
Lymph node involvement (N0 vs N+)	**0.021**	2.158	1.123–4.146	**0.044**	2.022	1.019–4.014
IVE (absence vs presence)	**< 0.001**	3.14	1.708–5.772	**< 0.001**	3.489	1.864–6.529
CA199(< 35 kU/L vs≥ 35 kU/L)	**0.004**	2.411	1.321–4.400	**0.013**	2.226	1.180–4.200
LMR (< 3.32 vs ≥ 3.32)	0.391	0.717	0.336–1.532	0.661	0.85	0.413–1.752
Blood glucose (mmol/L)	0.677	1.042	0.857–1.268	0.535	0.935	0.756–1.157
Neutrophil percentage (%)	0.209	1.061	0.967–1.163	0.258	1.049	0.966–1.139
Lymphocyte percentage (%)	0.289	1.06	0.952–1.182	0.443	1.038	0.943–1.144
coNLR-PDW 0	**0.002**			**< 0.001**		
coNLR-PDW 1	0.161	2.086	0.747–5.824	0.042	3.317	1.044–10.543
coNLR-PDW 2	0.004	5.197	1.659–15.933	< 0.001	12.619	3.576–44.521

Because NLR and PDW had a linear association with coNLR-PDW, it was not appropriate to group them for analysis using the COX multivariate model. Therefore, and to elucidate the role of NLR and PDW alone in predicting outcome of CRC patients, we replaced the coNLR-PDW index with NLR and PDW in the COX multivariate model. This modification yielded however similar results (Supplementay Table 4). In multivariate analysis, high PDW was related to inferior RFS (HR: 2.783, 95%CI: 1.600–4.843; *P* < 0.001) and OS (HR: 3.341, 95%CI: 1.892–5.899; *P* < 0.001). However, NLR was significantly associated with worse OS (HR: 5.179, 95%CI: 1.960–13.776; *P* = 0.001) but not with RFS (*P* = 0.239).

**Figure 2:**
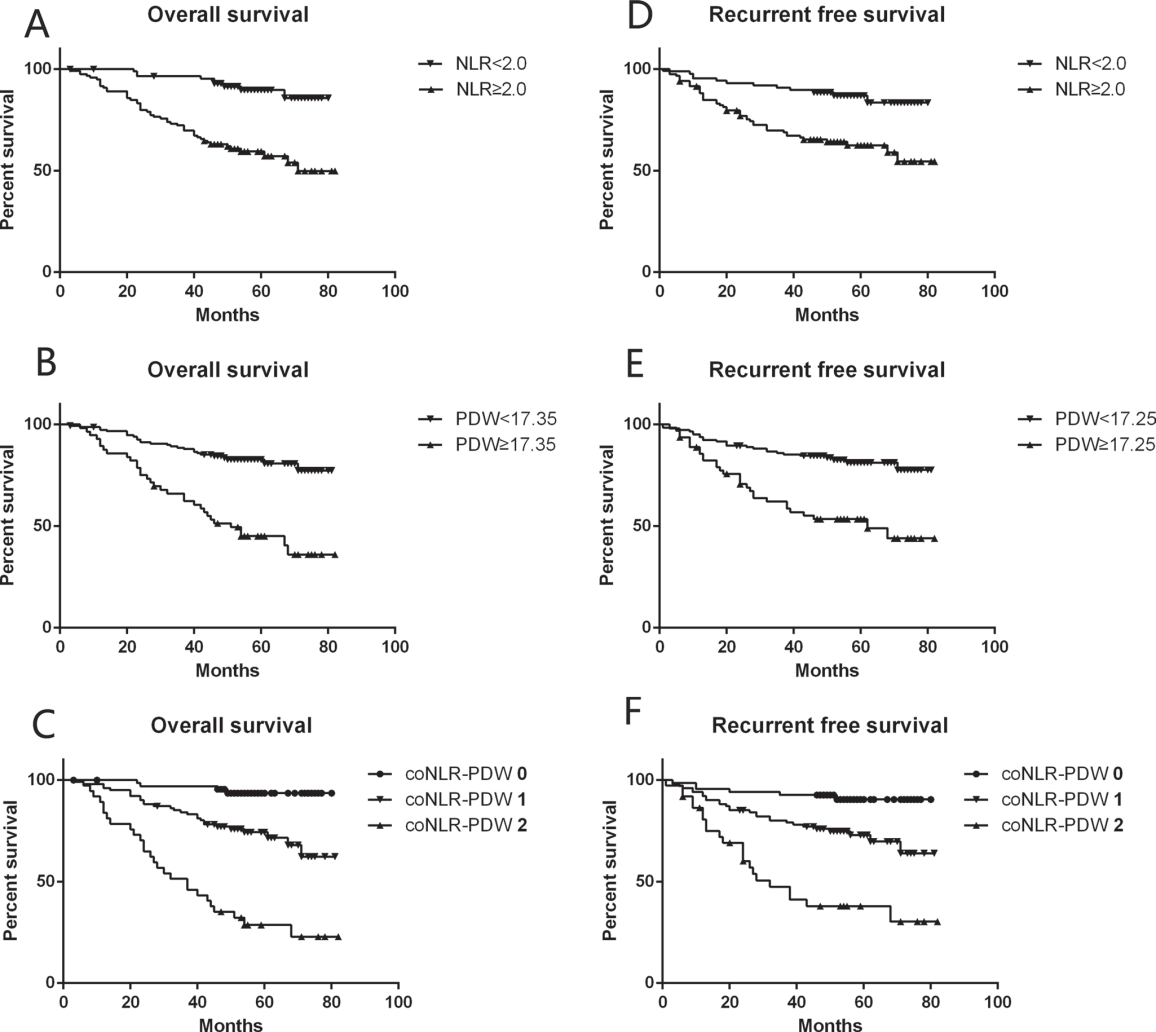
RFS and OS curves grouped by NLR, PDW, and coNLR-PDW (**A, B, C**) Patients with NLR ≥ 2.0, PDW ≥ 17.35%, and high coNLR-PDW scores had inferior OS (Log-rank *P* < 0.001 for all); (**D, E, F**) Patients with NLR ≥ 2.0, PDW ≥ 17.25%, and high coNLR-PDW scores had inferior RFS (Log-rank *P* < 0.001 for all).

Kaplan-Meier analysis and log-rank test were applied to assess for differences in RFS and OS between group pairs, defined by either NLR or PDW, or among the three groups determined by coNLR-PDW scores (Figure 2). Thus, the coNLR-PDW scoring system can effectively classify patients into three independent groups.

## DISCUSSION

Peripheral blood cell counts typically reflect the inflammatory status of patients, and can serve as useful predictors of prognosis in CRC and other types of cancers [7, 17]. In this study, we assessed the value of several inflammatory indices including neutrophil and lymphocyte percentages, platelet count, PDW, white blood cell ratios such as NLR and LMR, as well as a prognostic system that combines NLR and PDW, i.e. the coNLR-PDW index.

Inflammation and immunity play critical roles in cancer development and progression [18]. Cell-mediated immune responses are largely dependent on lymphocytes, and high numbers of tumor-infiltrating lymphocytes correlate to better prognosis [19, 20]. Neutrophils, in contrast, may promote a pro-tumoral environment through suppression of lymphocyte-mediated cytolysis and production of cytokines and chemokines that stimulate angiogenesis and tumor cell proliferation and metastasis [18, 21]. Tumor-associated macrophages, which arise from blood monocytes, contribute to tumor progression and metastasis by facilitating angiogenesis, matrix breakdown, and tumor cell motility [22]. Platelets have also been reported to act as chemoattractants, increasing the migration of ovarian cancer cells [23]. Thus, high NLR, PDW, or their combination seem to be related to a cancer-promoting environment, while a high LMR is related to active anticancer responses.

The importance of preoperative NLR as a predictor of CRC prognosis has been reported extensively [12, 24–33], yielding conclusions in agreement with our own results. However, the recommended NLR value, ranging from 2 to 5, was inconsistent. The recommended cut-off point in our study was 2.0, i.e. the same value used by Liu et al. as the upper limit in healthy controls [25], and lower than the NLR threshold defined in many previous studies. The reason may be the small number of patients enrolled in our study and the exclusion of metastasis cases, because late-stage cancer may positively relate to more severe inflammation. In accord with Jankova et al. [24], we found elevated NLR to be an independent adverse predictor of OS, but not RFS, in patients with non-metastatic CRC. Though both neutrophil and lymphocyte percentages were linked to prognosis in univariate analysis, no significant association was detected by multivariate analysis in our study.

Preoperative LMR as a valuable predictor of prognosis in patients with colon cancer was first demonstrated by Stotz et al. [9]. Elevated monocyte count (≥ 545/mm3) or monocyte percentage (> 7%) in peripheral blood is an independent predictor of poor prognosis in patients with hepatocellular carcinoma after hepatic resection [34, 35]. However, we did not find any significant prognostic value for monocyte abundance in our CRC patients, and a LMR with a recommended cut-off value of 3.32, as per the ROC curve, was also not significant in multivariate analysis. Monocytes play a controversial role in cancer development and progression, as they can promote or restrict cancer growth depending on such factors as cancer type and stage [36].

Larger platelets have more granules and greater secretory capacity than smaller ones, and are activated more readily. Thus, platelet size correlates with platelet activity and the latter can be inferred by PVI such as MPV and PDW. PVI is useful in the setting of some diseases such as hematology disorders, vascular disease, coronary artery disease, venous thromboembolism, and inflammatory disease, among others [16, 37]. The present study was one of the few that assessed the value of PDW in patients with CRC. We found that PDW, but not MPV or platelet count, satisfactorily predicted prognosis in patients with CRC. Interestingly, although patients with higher PDW had, accordingly, significantly higher MPV values, coagulation-related indices, including activated partial thromboplastin time (APTT), prothrombin time (PT), and the international normalized ratio (INR) were similar in high and low PDW patients, and did not predict prognosis either. This indicated that a high PDW could reflect, at least partly, the level of activated platelets, which is closely related to either inflammation or tumor invasion and metastasis, but does not necessarily imply impaired coagulation.

The coNLR-PDW system combines two inflammation-related indices, NLR and PDW, so it may reflect the systemic inflammatory response (SIR) status more comprehensively, and is arguably a superior predictor of prognosis. To the best of our knowledge, this is the first study investigating the association of the coNLR-PDW index with prognosis in CRC patients. In accord with our expectation, such index can effectively classify the patients into three groups and served as a strong, independent prognostic factor for RFS and OS in patients with non-metastatic CRC.

Besides coNLR-PDW, IVE and lymph node involvement were also robust outcome predictors, which was consistent with the results of our previous study [38]. However, NLR, PDW, and coNLR-PDW were all correlated with IVE by χ^2^-test. This association may be related to the expression of vascular endothelial growth factor (VEGF) and its role in neoangiogenesis and tumor cell vasculogenesis, two pathophysiological processes potentially conducive to IVE [38, 39]. Serum VEGF is correlated with prognosis in various cancers, and circulating VEGF resides mainly in platelets and neutrophils [40, 41]. After being activated, platelets and neutrophils release VEGF locally, which could promote cancer invasion and metastasis. This may partly explain the relationship between inflammatory indices and IVE, although our data suggest that the coNLR-PDW index is a stronger predictor of prognosis in patients with non-metastatic CRC.

In conclusion, we demonstrated that preoperative elevated PDW and a high coNLR-PDW score independently predicted worse RFS and OS in non-metastatic CRC patients, while a high NLR was independently related to inferior OS, but not to RFS. In addition, IVE was also an independent prognostic factor and was associated with the inflammatory indices mentioned above. Our results highlight the usefulness of the coNLR-PDW index as prognostic marker of non-metastatic CRC outcome; its assessment, along with that of other relevant prognostic indicators such as IVE, might contribute to establishing more individualized regimes for patients undergoing tumor resection surgery.

## MATERIALS AND METHODS

### Patients and clinical follow-up

We conducted a retrospective review of a database comprising 206 patients who had undergone curative surgery for CRC between January 2009 and December 2011 at a single institution (Xiangya Hospital, Hunan, Changsha, China). The inclusion criteria required that patients had received curative surgery for CRC, presenting histologically confirmed non-metastatic (including TNM I, II, or III) colorectal cancer on post-surgery analysis. Disease stage was established in accordance with the AJCC7th classification. The exclusion criteria included: neoadjuvant chemotherapy or other anti-cancer therapies before surgery; drug use, including NSAIDs, before surgery; colorectal cancer with intestinal perforation or obstruction; vascular disorder or inflammation-related diseases; and incomplete clinicopathological data. 119 out of 206 patients received adjuvant chemotherapy after the surgery. Among the 119 patients, there were 3 out of 37 with TNM I (CapeOX, 2; FOLFOX6, 1), 39 out of 67 with TNM II (CapeOX, 13; FOLFOX6, 19; FOLFOX4, 6; OFL, 1), and 77 out of 102 with TNM III (CapeOX, 13; FOLFOX6, 41; FOLFOX4, 22; OFL, 1). After surgery, we conducted telephone follow-ups every three months in the first year, and twice per year during subsequent years. Follow-up investigations included clinical check-up, laboratory (including blood routine examination and cancer-related marker analysis, such as CEA and CA 199, every 3–6 months), radiological assessment (abdomen and chest computed tomography, every 6–12 months) and colonoscopy every year if possible. All patients were followed up from 3 to 82 months after surgical treatment and the last date of follow-up was November 15, 2015. RFS was defined as the interval from radical surgery to recurrence, metastasis, or death, whichever occurred first. OS was defined as the interval from radical surgery to mortality, or it was censored at the last known date that the patient was alive.

### Clinicopathological data

Blood laboratory measurements were carried out within 7 days before surgery. All patient-related data were retrieved from the medical record database, including blood test values, MPV, PDW, some biochemical indicators such as serum albumin levels, serum levels of carcinoembryonic antigen (CEA) and carbohydrate antigen 199 (CA199), as well as demographic information and postoperative pathological results. NLR was calculated for each patient as the absolute neutrophil count divided by the absolute lymphocyte count. LMR was calculated as the absolute lymphocyte count divided by the absolute monocyte count. PDW was computed automatically as the coefficient of variation of the average volume of the platelet population.

### ROC curve analysis

The receiver operating characteristic (ROC) curve analysis was used to assess optimal cut-off values of preoperative NLR, LMR, and PDW for RFS and OS analyses. Based on the sensitivity and specificity values, for both RFS and OS the recommended cut-off values were 2.0 for NLR and 3.32 for LMR. For PDW, recommended cut-off values of 17.25% and 17.35% were defined for RFS and OS, respectively.

### NLR and PDW scoring method

We defined the scores of NLR as 1 or 0 when patients had a high (≥ 2.0) or a low (< 2.0) NLR, respectively. Similarly, the PDW scores were 1 or 0 when patients had, respectively, a high (≥ 17.35%) or a low (< 17.35%) PDW. The combined score (coNLR-PDW) was defined as follows: patients with both high NLR and high PDW were assigned a score of 2, and patients scoring high for only one parameter, or low for both, were assigned a score of 1 or 0, respectively.

### Statistical analysis

Differences among the groups were analyzed by χ2-test. The *t-test* was used to analyze the differences between means of two groups, and one-way ANOVA or Welch's test was used to compare three groups. Data are presented as mean±s.d. Univariate analysis was performed to evaluate clinical characteristics including NLR, PDW, LMR, and other factors related to RFS and OS. In multivariate Cox regression analysis, the model was adjusted for prognostic clinicopathological factors in univariate analysis. Hazard ratios (HRs) estimated from the Cox regression analysis were reported as relative risks with corresponding 95% confidence intervals. Survival curves were made by using the Kaplan–Meier method and compared by the log-rank test. All statistical analyses were performed using the Statistical Package for Social Sciences version 22.0 (SPSS Inc., Chicago, IL, USA). A two-sided *P* < 0.05 was considered statistically significant.

## SUPPLEMENTARY MATERIALS AND TABLES


