# Supplementary material for: Significance of inflammation-based indices in the prognosis of patients with non-metastatic colorectal cancer
Source: Oncotarget. 2017 Apr 1;8(28):45178–89. doi: 10.18632/oncotarget.16774 (PMC5542176; doi:10.18632/oncotarget.16774)
Supplement: Supplementary file 1 [file oncotarget-08-45178-s001.pdf]

Significance of inflammation-based indices in the prognosis of patients with non-metastatic colorectal cancer

Supplementary Materials

Supplementary Table 1: Baseline demographic and clinical characteristics of patients

Characteristics		Patients (n = 206)
Age	≥ 60	93 (45.1%)
	< 60	113 (54.9%)
Gender	Male	125 (60.7%)
	Female	81 (39.3%)
Location	Colon	106 (51.5%)
	Rectum	100 (48.5%)
Stage	TNM I	37 (18.0%)
	TNM II	67 (32.5%)
	TNM III	102 (49.5%)
Differentiation	Well	162 (78.6%)
	Poor	44 (21.4%)
Tumor invasion depth	T1–2	52 (25.2%)
	T3–4	154 (74.8%)
Lymph node involvement	N0	104 (50.5%)
	N+	102 (49.5%)
IVE	Absence	154 (74.8%)
	Presence	52 (25.2%)
Diameter	≥ 5 cm	119 (57.8%)
	< 5 cm	87 (42.2%)
CEA	≥ 5 ng/ml	50 (24.3%)
	< 5 ng/ml	156 (75.7)
CA199	≥ 35 kU/L	30 (14.6%)
	< 35 kU/L	176 (85.4%)
NLR	≥ 2.0	119 (57.8%)
	< 2.0	87 (42.2%)
LMR	≥ 3.32	120 (58.3%)
	< 3.32	86 (41.7%)
Adjuvant chemotherapy	Yes	119 (57.8%)
	No	87 (42.2%)
Laboratory parameters		mean (range)
Albumin (g/dl)		38.2 (26.7–50.8)
Blood glucose (mmol/L)		5.00 (2.04–10.81)
Hemoglobin (g/L)		112 (16–158)
Platelet count ($\times 10^3$/ml)		242 (57–702)
RBC count ($\times 10^6$/ml)		3.99 (2.01–5.66)
WBC count ($\times 10^3$/ml)		6.11 (2.58–14.88)
Neutrophil percentage (%)		60.1 (28.0–92.4)
Lymphocyte percentage (%)		27.7 (1.0–57.0)
Monocyte percentage (%)		8.0 (0.8–18.0)
MPV (fl)		8.8 (6.2–15.9)
PDW (%)		16.9 (7.0–22.5)
APTT (sec)		36.5 (23.2–46.7)
PT (sec)		12.8 (11.0–16.6)
INR		0.98 (0.81–1.35)
NLR		3.3 (0.5–132.0)
LMR		3.90 (0.11–25.27)

Supplementary Table 2: Relationships between clinical characteristics and PDW

Parameters		PDW (%)		*P*
		PDW < 17.25 (*n* = 143)	PDW ≥ 17.25 (*n* = 63)	
Age	≥ 60	63	30	0.636
	< 60	80	33	
Gender	Male	82	43	0.140
	Female	61	20	
Location	Colon	72	34	0.632
	Rectum	71	29	
Stage	TNM I	28	9	0.060
	TNM II	52	15	
	TNM III	63	39	
Differentiation	Well	112	50	0.866
	Poor	31	13	
Tumor invasion depth	T1–2	35	17	0.703
	T3–4	108	46	
Lymph node involvement	N0	80	24	0.018
	N+	63	39	
IVE	Absence	113	41	0.034
	Presence	30	22	
Diameter	≥ 5 cm	86	33	0.299
	< 5 cm	57	30	
CEA	≥ 5 ng/ml	34	16	0.803
	< 5 ng/ml	109	47	
CA199	≥ 35 kU/L	18	12	0.226
	< 35 kU/L	125	51	
LMR	≥ 3.32	89	31	0.081
	< 3.32	54	32	
NLR	≥ 2.0	79	40	0.270
	< 2.0	64	23	
Adjuvant chemotherapy	Yes	88	31	0.099
	No	55	32	

Supplementary Table 3: Relationships between clinicolaboratory characteristics and PDW

Parameters	PDW (%)		*P*
	PDW < 17.25 (*n* = 143)	PDW ≥ 17.25 (*n* = 63)	
Age (year)	57.0 ± 12.7	57.3 ± 12.6	0.871
Albumin (g/dl)	38.1 ± 3.7	38.3 ± 4.2	0.711
Blood glucose (mmol/L)	5.0 ± 1.0	5.0 ± 1.1	0.862
Platelet count (×10^3/ml)	252.8 ± 94.4	217.3 ± 81.4	**0.010**
Hemoglobin (g/L)	112.3 ± 22.8	112.1 ± 22.1	0.948
WBC count (×10^3/ml)	6.2 ± 2.0	5.8 ± 2.1	0.177
RBC count (×10^6/ml)	4.0 ± 0.5	4.0 ± 0.6	0.346
Neutrophil percentage (%)	59.3 ± 9.5	61.9 ± 6.4	0.072
Lymphocyte percentage (%)	28.5 ± 8.5	25.7 ± 8.7	**0.032**
Monocyte percentage (%)	7.9 ± 2.4	8.3 ± 2.5	0.280
MPV (fl)	8.3 ± 1.4	9.8 ± 1.7	**< 0.001**
PT (sec)	12.8 ± 0.8	12.9 ± 0.8	0.173
INR	0.97 ± 0.08	0.99 ± 0.08	0.095
APTT (sec)	36.2 ± 4.0	37.1 ± 3.4	0.134
NLR	2.6 ± 3.1	4.8 ± 16.4	0.286
LMR	4.1 ± 2.6	3.5 ± 1.6	0.067
PDW (%)	16.4 ± 1.2	18.0 ± 1.0	**< 0.001**

Supplementary Table 4: Multivariate analysis in relation to RFS and OS

Parameters	RFS			OS		
	P	HR	95% CI	*P*	HR	95% CI
Location (colon vs rectum)	NA			**< 0.001**	2.959	1.636–5.350
Tumor invasion depth (T1–2 vs T3–4)	0.262	1.606	0.701–3.678	NA		
Lymph node involvement (N0 vs N+)	**0.029**	2.082	1.077–4.025	**0.034**	2.105	1.057–4.194
IVE (absence vs presence)	**< 0.001**	3.228	1.759–5.922	**< 0.001**	3.524	1.895–6.554
CA199 (< 35 kU/L vs ≥ 35 kU/L)	**0.004**	2.404	1.314–4.397	**0.013**	2.227	1.181–4.199
LMR (< 3.32 vs ≥ 3.32)	0.359	0.702	0.329–1.497	0.652	0.849	0.417–1.729
NLR (< 2.0 vs ≥ 2.0)	0.239	1.76	0.687–4.510	**0.001**	5.197	1.960–13.776
PDW (< 17.35 vs ≥ 17.35)	**< 0.001**	2.783	1.600–4.843	**< 0.001**	3.341	1.892–5.899
Blood glucose (mmol/L)	0.643	1.048	0.860–1.276	0.531	0.934	0.755–1.156
Neutrophil percentage (%)	0.191	1.064	0.970–1.167	0.376	1.039	0.955–1.131
Lymphocyte percentage (%)	0.318	1.055	0.949–1.173	0.416	1.041	0.944–1.149
